# The complete mitochondrial genome of *Bombyx lemeepauli* (Lepidoptera: Bombycidae) and its phylogenetic relationship

**DOI:** 10.1080/23802359.2017.1361360

**Published:** 2017-08-08

**Authors:** Shuang-Qing Liu, Xing-Shi Gu, Xing Wang

**Affiliations:** a College of Plant Protection, Hunan Agricultural University, Changsha, Hunan, China;; b Hunan Provincial Key Laboratory for Biology and Control of Plant Diseases and Insect Pests, Hunan Agricultural University, Changsha, Hunan, China

**Keywords:** *Bombyx lemeepauli* Lemée, mitochondrial genome, evolutionary relationships

## Abstract

The complete mitochondrial genome (mitogenome) of *Bombyx lemmepauli* Lemée has been sequenced with 15,801 bp in length (Genbank no. KY620270), and has a base composition of A (43.17%), G (7.40%), C (11.90%), and T (37.86%). Similar to other bombyciod species, it contains a typically conserved structure including 13 protein-coding genes (PCGs), 22 transfer RNA genes, 2 ribosomal RNA genes, and an A + T-rich region. Excepting *cox1* started with CGA, the start codons of the other 12 PCGs were ATN. Eleven of the 13 PCGs ended with TAA, expect for *cox1* and *cox2*, which ended with a single T. The complete mitogenome sequence provided here would be useful for further understanding the evolutional position of *B. lemeepauli*, which is a key species to relate the famous resource insect *B. mori* and the important insect pest *Rondotia menciana* (Lepidoptera: Bombycidae).

The silkworm moth, *Bombyx lemeepauli* Lemée (Lepidoptera: Bombycidae) forming a typical white semilunar fasciae on a gray ground color, is endemic to the Himalayan region and is a key species to relate the famous resource insect *B. mori* and the important insect pest *Rondotia menciana* (Wang et al. 2015). To ensure and investigate the deeper phylogenetic position, the complete mitochondrial genomes (mitogenome) of *B. mori* and *R. menciana* have been determined (Liu et al. 2013; Kong and Yang 2015). However, the *B. lemeepauli* mitogenome has not been reported.

Here, the mitogenome of *B. lemeepauli* which adult was collected from Jialing River source scenic spot in Qinling Mountains in China with its genomic DNA stored in Hunan Agricultural University, was sequenced and characterized. Eleven pairs of primer (Gu et al. 2016) were used to amplify the complete mitogenome. The fragments were proof-read by the software Geneious version 8.1.2 (Kearse et al. 2012), and the automatic annotation was performed by the online-program MITOS (http://mitos.bioinf.uni-leipzaig.de) (Bernt et al. 2013) with the annotated genes deposited in GenBank (accession number KY620270). The entire mitogenomes of 25 bombycoid species as ingroups and two geometrid species as outgroups were obtained from NCBI. The conserved regions of the putative amino acids were filtrated by the program Gblock 0.91b with default settings (Castresana 2000). The phylogenetic tree reconstructed by Maximum Likelihood (ML) with 1000 replications and Bayesian Inference (BI) with running for 10,000,000 generations.

The whole mitogenome of *B. lemeepauli* has a closed circular with 15,801 bp in length, and encoded 37 genes, including 13 PCGs (11,130 bp in total), 22 tRNA genes, 2 rRNA genes, and a putative A + T-rich region. The 209-bp intergenic spacer sequences were observed which the regions dispersed in 17 pairs of neighboring genes with the length varying from 1 to 56 bp. There were 8 overlapping nucleotide fragment in the mitogenome with the longest region between *trnL* (CUN) and *rrnL* (−25bp). Excepting *cox1* started with CGA, the start codons of the other 12 PCGs were ATN. Regarding the stop codons, eleven PCGs stopped at TAA, except *cox1* and *cox2*. Twenty-two tRNA genes range from 65 to 72 bp in length and display a high AT content of 81.64%. The two rRNA genes, *rrnL* and *rrnS* both mapped on the N-strand, were 1392 bp and 791 bp in length.

The evolutionary relationships among the Bombycoidea were reconstructed and the topological structures of the ML and BI trees were identical (Figure 1). The bombycoid was strongly supported as a monophyletic group by the bootstrap value of 100% and the posterior probability of 1.00, and the relationship within this superfamily were Lasiocampidae + (Saturniidae + Endromidae) + (Bombycidae + Sphingidae)). Furthermore, the phylogenetic position of *B. lemeepauli* among bombycid were *T. daii* + (*R. menciana* + (*B. lemeepauli* + (*B. huttoni* + (*B. mandarina* +* B. mori*) which was supported as a monophyletic clade by a bootstrap value of 100% and a posterior probability of 1.00. The evolutionary relationships of these analyzed species are consistent with previously reported results based on morphological characters (Wang et al. 2015). The newly determined mitogenome will help to understand the evolution of the silk moths.

**Figure 1.**
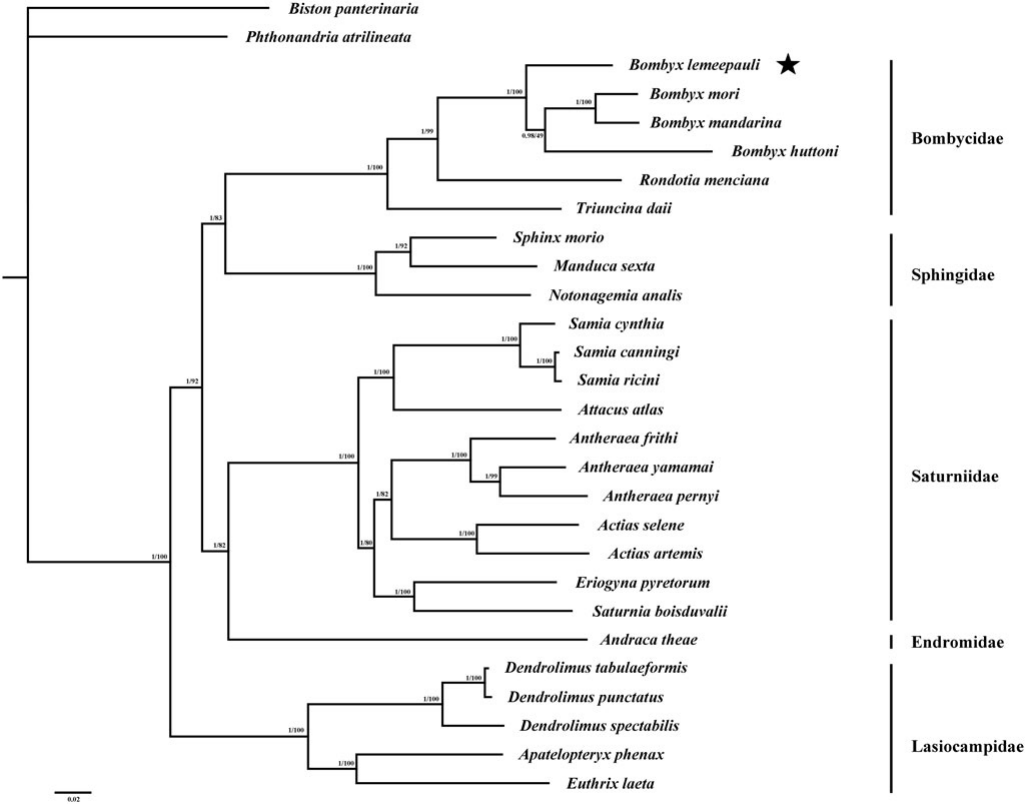
Bayesian inference and Maximum likelihood phylogram constructed using 13 PCGs of mitogenomes with partitioned models. Numbers above each node indicates the ML bootstrap support values and the BI posterior probability. All the species’ accession numbers in this study are listed as below: *Actias artemis* KF927042, *Actias selene* NC_018133, *Andraca theae* KX365419, *Antheraea frithi* NC_027071, *Antheraea pernyi* NC_004622, *Antheraea yamamai* NC_012739, *Apatelopteryx phenax* KJ508055, *Attacus atlas* NC_021770, *Biston panterinaria* NC_020004, *Bombyx huttoni* NC_026518, *B. lemeepauli* KY620270, *Bombyx mandarina* NC_003395, *Bombyx mori* NC_002355, *Dendrolimus punctatus* NC_027156, *Dendrolimus spectabilis* NC_025763, *Dendrolimus tabulaeformis* NC_027157, *Eriogyna pyretorum* NC_012727, *Euthrix laeta* NC_031507, *Manduca sexta* NC_010266, *Notonagemia analis* KU934302, *Phthonandria atrilineata* NC_010522, *Rondotia menciana* NC_021962, *Samia canningi* NC_024270, *Samia cynthia* KC812618, *Samia ricini* NC_017869, *Saturnia boisduvalii* NC_010613, *Sphinx morio* NC_020780, *Triuncina daii* KY091643.
